# EGBMMDA: Extreme Gradient Boosting Machine for MiRNA-Disease Association prediction

**DOI:** 10.1038/s41419-017-0003-x

**Published:** 2018-01-05

**Authors:** Xing Chen, Li Huang, Di Xie, Qi Zhao

**Affiliations:** 1 School of Information and Control Engineering, China University of Mining and Technology, Xuzhou, 221116 China; 2 Business Analytics Centre, National University of Singapore, Singapore, 119613 Singapore; 3 School of Mathematics, Liaoning University, Shenyang, 110036 China; 4 Research Center for Computer Simulating and Information Processing of Bio-Macromolecules of Liaoning Province, Shenyang, 110036 China

## Abstract

Associations between microRNAs (miRNAs) and human diseases have been identified by increasing studies and discovering new ones is an ongoing process in medical laboratories. To improve experiment productivity, researchers computationally infer potential associations from biological data, selecting the most promising candidates for experimental verification. Predicting potential miRNA–disease association has become a research area of growing importance. This paper presents a model of Extreme Gradient Boosting Machine for MiRNA-Disease Association (EGBMMDA) prediction by integrating the miRNA functional similarity, the disease semantic similarity, and known miRNA–disease associations. The statistical measures, graph theoretical measures, and matrix factorization results for each miRNA-disease pair were calculated and used to form an informative feature vector. The vector for known associated pairs obtained from the HMDD v2.0 database was used to train a regression tree under the gradient boosting framework. EGBMMDA was the first decision tree learning-based model used for predicting miRNA–disease associations. Respectively, AUCs of 0.9123 and 0.8221 in global and local leave-one-out cross-validation proved the model’s reliable performance. Moreover, the 0.9048 ± 0.0012 AUC in fivefold cross-validation confirmed its stability. We carried out three different types of case studies of predicting potential miRNAs related to Colon Neoplasms, Lymphoma, Prostate Neoplasms, Breast Neoplasms, and Esophageal Neoplasms. The results indicated that, respectively, 98%, 90%, 98%, 100%, and 98% of the top 50 predictions for the five diseases were confirmed by experiments. Therefore, EGBMMDA appears to be a useful computational resource for miRNA–disease association prediction.

## Introduction

Emerging as a post-transcriptional regulator of gene expressions, microRNAs (miRNAs) are short non-coding RNAs of about 22 nucleotides in length found in a wide range of species, including viruses, plants, and animals^1–3^. Their regulatory mechanism involves base-pairing to sites within the 3′ untranslated region (UTR) of their target messenger RNAs (mRNAs)^4,5^. MiRNAs influence most cellular pathways, including cell proliferation, differentiation, death, and signal transduction^4,6,7^. Deficiencies or excesses in miRNA expressions are correlated to abnormal biological processes and hence human diseases^8^. In particular, miRNA aberrances have a strong association with various cancers and cancer-related processes^9,10^. Chronic lymphocytic leukemia was one of the first human cancers detected to be related to dysregulation of miRNAs^11^. MiR-15 and miR-16 located at chromosome 13q14 are frequently deleted in more than half of B cell chronic lymphocytic leukemias. Since then, more associations between miRNAs and cancers have been discovered. For instance, the commonly found dysregulation of miR-200a, b, and c carries a potential role in the pathogenesis and progression of conjunctival MALT Lymphoma^12^. Another example is an upregulated expression of miR-183 in prostate cancer cells and that inhibiting it may benefit the prostate cancer treatment^13^. Well-known databases storing these known associations between miRNAs and diseases (not just cancers) include HMDD v2.0^14^, dbDEMC^15^, and miR2Disease^16^. But even when combined, the databases are by no means exhaustive; continuously there are experiments carried out and literatures published to support new associations. The major motivation of identifying novel disease-related miRNAs is to facilitate diagnosis, progression, prognosis, and treatment of complex diseases^8,17^. With the aid of the large amount of available biological data, researchers develop computational models to prioritize potential disease-related miRNAs in terms of prediction scores and experiment on ones with the highest association likelihood. This approach reduces the number of futile experiments and saves researchers’ time and cost.

The past few years have witnessed significant progresses in developing prediction models for potential disease–miRNA associations. The models broadly fall into the network analysis category or the machine learning category. Most computational models were developed under the assumption that functionally similar miRNAs tend to be connected with phenotypically similar diseases^18–20^. Jiang et al.^21^ presented one of the initial models for predicting disease-related miRNAs. The miRNA functional similarity network, the disease phenotype similarity network, and the known disease–miRNA association network were integrated in the model and a discrete probability distribution named hypergeometric was used to score the potential miRNA–disease associations. The drawback of the model was that it only considered the neighbor information of each miRNA in the scoring system. Incorporating global network similarity information into the model would increase its accuracy. In an HDMP model proposed by Xuan et al.^22^, the miRNA–disease associations were combined with the miRNA functional similarity, the disease semantic similarity, and the disease phenotype similarity. Considering each miRNA’s *k* most similar neighbors into the calculations yielded an improved accuracy compared to previous models, because higher weights were assigned to miRNAs in the same cluster or family. Nevertheless, HDMP failed to make predictions for new diseases without known related miRNAs. Making use of global similarity measures, not solely local similarity information, would overcome the weakness of the model. Chen et al.^23^ presented a Random Walk with Restart model named RWRMDA, seeking putative disease-related miRNAs with similar functions to known disease-related miRNAs. The model achieved a satisfactory accuracy via the application of global similarity measures, but was still unable to work for new diseases without any known related miRNAs. Later, Xuan et al.^24^ further introduced a Random Walk model named MIDP in which labeled nodes were given higher transition weights than unlabeled nodes. The model effectively exploited the prior information of nodes and various ranges of topologies, and by controlling the restart rate it alleviated the negative effect of noisy data. In addition, the walk on the disease–miRNA network was extended so that candidates for diseases without any known related miRNAs could be predicted. Chen et al.^25^ also made such predictions possible and reliable by releasing a novel model called WBSMDA. Not only did the model use the miRNA functional similarity, disease semantic similarity, and miRNA–disease associations but also it calculated Gaussian interaction profile kernel similarity for diseases and miRNAs. Another HGIMDA model presented by Chen et al.^26^ had the same model inputs but integrated the diseases/miRNAs similarities with Gaussian interaction profile kernel similarities in a slight different manner from WBSMDA. The new similarity networks for diseases and miRNAs, together with the miRNA–disease association network, were further combined into a heterogeneous graph. An iterative procedure was implemented on the graph to infer potential associations between a miRNA and a disease, even if they had no known associations. A more recent MCMDA model was published by Li et al.^27^. A matrix completion algorithm was adopted in the model and of a high efficiency in updating the lowly ranked miRNA–disease matrix. Unlike some previous models requiring negative associations, MCMDA only depended on the known miRNA–disease associations.

Researchers have also developed models based on various types of association networks, not just miRNA–disease association network. Shi et al.^28^ carried out hierarchical clustering on the known miRNA–disease association network and reached a conclusion that a disease is more likely to connect with miRNAs whose target genes are related to that disease. Based on this, they proposed a Random Walk model on a protein–protein interaction network. Mork et al.^29^ devised an miRPD model combining protein–disease interactions and protein–miRNA interactions as predictors and outputting potential disease-related miRNAs and disease-related proteins. The intension of involving proteins in the output was to facilitate the protein link between miRNAs and diseases, allowing for more explicit design of verification experiments. Pasquier et al.^30^ developed an MiRAI model that concatenated five distinct matrices: (1) the miRNA–disease association matrix, (2) the miRNA-neighbor association matrix, whose edges were weighted by the genomic distance between two miRNAs, (3) the miRNA–target association matrix, (4) the miRNA–word association matrix, whose edges were weighted by the TF-IDF weighting scheme on the associated documents for the investigated miRNAs, and (5) the miRNA–family association matrix. Then, the large matrix as a result of the concatenation was input to Singular Value Decomposition for dimensionality reduction. The cosine similarity between an miRNA in the miRNA space and a disease in the disease space was the association score for this miRNA–disease pair.

As an alternative to the aforementioned network analysis-based models, various machine learning-based models have emerged to make sound predictions. Xu et al.^31^ performed feature extraction based on the topology information of a heterogeneous miRNA-target dysregulated network (MTDN). The network was the combination of miRNA–target interactions and the expression profiles of miRNAs and mRNAs in tumor and non-tumor tissues. A support vector machine classifier was constructed in MTDN to separate positive miRNA–disease associations from negative ones. A limitation persisting in the model, however, is that determining negative associations is difficult and even impossible^26^. Therefore, the performance of the model could be unstable given an inaccurate selection of negative samples. To address the problem, Chen et al.^32^ proposed an RLSMDA model based on semi-supervised learning framework. Notably, no negative samples were required to fit the model. Subsequently, Chen et al.^33^ published an RBMMMDA model where a two-layered (with visible and hidden units) undirected miRNA–disease graph was built according to restricted Boltzmann machine (RBM). RBMMMDA could predict both novel miRNA–disease associations and the corresponding association types, which was unique to other models.

Over the time, the prediction accuracy of computational models for predicting miRNA–disease associations is continuously increasing. In search of a superior model over previous ones, we developed a machine learning-based model, Extreme Gradient Boosting Machine for MiRNA-Disease Association prediction (EGBMMDA). The input to the model was a feature vector for the miRNA–disease pair (*m*(*i*),*d*(*j*)), obtained from feature extraction on the miRNA functional similarity, the disease semantic similarity, and the known miRNA–disease associations. The vector covered statistical measures, graph theoretical measures, and matrix factorization results for (*m*(*i*),*d*(*j*)). The model’s output was an association score for this pair. Global and local leave-one-out cross-validations (LOOCVs), fivefold cross-validation, and five case studies were carried out to evaluate the performance of EGBMMDA. HMDD v2.0^14^ was used as the training database for the model throughout the evaluation (except for the fifth case study that was based on the older version of HMDD). EGBMMDA consistently outperformed previous models in every cross-validation and a large proportion of the predicted miRNA–disease associations were experimentally confirmed in each case study. To our knowledge, no existing computational models make use of decision trees to predict novel miRNA–disease associations, and to date, EGBMMDA is one of the very few models that achieved a global LOOCV AUC greater than 0.9.

## Results

### Performance evaluation

**Fig. 1:**
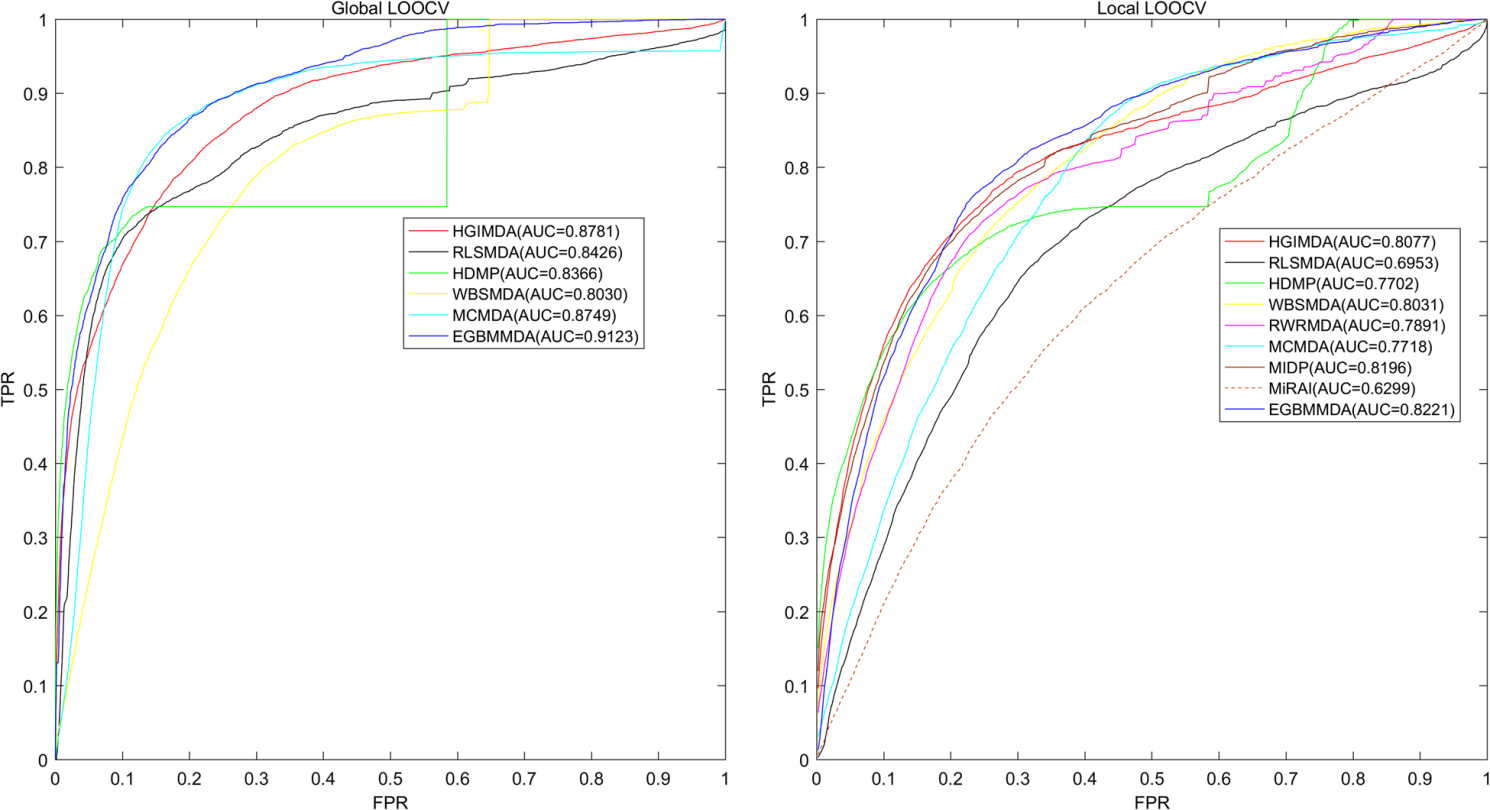
Performance comparisons between EGBMMDA and eight previous disease–miRNA association prediction models (RLSMDA, MiRAI, MCMDA, HGIMDA, WBSMDA, MIDP, RWRMDA, and HDMP) in terms of ROC curve and AUCs based on local and global LOOCV, respectively. As a result, EGBMMDA achieved AUCs of 0.9123 and 0.8221 in the global and local LOOCV, surpassing all the previous models

The performance of EGBMMDA was evaluated by LOOCV and fivefold cross-validation on the known miRNA–disease association dataset retrieved from HMDD v2.0 (ref. 14). The database recorded 383 diseases and 495 miRNAs, which constituted 5430 known associations. We implemented LOOCV under global and local frameworks, plotted receiver operating characteristics (ROC) curves, and used area under the ROC curve (AUC) as the evaluation metric. As illustrated in Fig. 1, EGBMMDA achieved AUC of 0.9123 in global LOOCV and AUC of 0.8221 in local LOOCV, reflecting an effective prediction performance of the model. Figure 1 also shows that EGBMMDA consistently outperformed the models introduced in previous studies^22–27,30,32^. In global LOOCV, MCMDA, HGIMDA, WBSMDA, RLSMDA, and HDMP obtained AUCs of 0.8749, 0.8781, 0.8030, 0.8426, and 0.8366, respectively; in local LOOCV, they exhibited AUCs of 0.7718, 0.8077, 0.8031, 0.6953, and 0.7702. RWRMDA and MIDP were not included in global LOOCV comparison, because they were based on random walk that was a local approach and could not simultaneously make predictions for all diseases. In addition, global LOOCV was not applicable to MiRAI, either, because the association scores given by this model were highly positively correlated with the seed count (that is, the number of known associated miRNAs) of a disease. For a disease with more associated miRNAs, the association scores for its candidate miRNAs tended to be higher, and vice versa. Therefore, the associations scores obtained for different diseases were not comparable. The AUCs in local LOOCV for RWRMDA, MIDP, and MiRAI were 0.7891, 0.8196, and 0.6299, respectively. MiRAI had a low AUC because the core to this method was collaborative filtering that suffers from the data sparsity problem. Our training dataset was sparse; it contained 383 diseases, of which the majority were associated with only a few miRNAs. MiRAI became less performative when evaluated on our dataset than when tested on 83 diseases with at least 20 known associated miRNAs in the literature^30^. Since the AUCs for previous models were lower than that for EGBMMDA, we could consider the latter model as an advancement in the exploration of reliable miRNA–disease association prediction models. As for the fivefold cross-validation result, the model achieved an AUC of 0.9048 ± 0.0012. The 0.9048 mean value surpassed MCMDA’s 0.8767, HDMP’s 0.8342, and WBSMDA’s 0.8185, and the 0.0012 standard deviation proved the stability of EGBMMDA.

### Case studies

We carried out five case studies to demonstrate how accurately EGBMMDA could predict novel miRNA–disease associations. In all five case studies, a high proportion of the potential disease-related miRNAs were experimentally confirmed, implying that EGBMMDA made reliable predictions. The first three cases studies concerned with Colon Neoplasms (CN), Lymphoma, and Prostate Neoplasms (PN), and known miRNA–disease associations from HMDD v2.0 were used as the training samples for the model. All candidate miRNAs for the investigated disease were ranked by their association scores. A candidate miRNA was defined as a miRNA unassociated with the investigated disease according to HMDD v2.0. Subsequently, the top 10 and 50 candidates were used as the prediction lists and validated against another two prominent miRNA–disease association databases dbDEMC^15^ and miR2Database^16^ as well as other experimental literatures. Because only candidate miRNAs were ranked and validated, there was no overlap between the training samples and the prediction lists.

CN is most frequently diagnosed in developed countries. It has been estimated that in 2017 in the United States there will be 135,430 newly diagnosed CN cases and 50,260 deaths caused by CN^34^. In CN tumor cells, dysregulation of miRNAs has been observed to have the potential of serving as diagnostic biomarkers for CN^35^. Current candidate biomarkers for CN include miR-126 and miR-145 that inhibit the growth of CN cells by targeting the phosphatidylinositol 3-kinase signaling and the insulin receptor substrate-1, respectively^36,37^. But they may not be sufficient. Novel sensitive biomarkers are increasingly in demand and can be useful for improving CN detections^38^. Thus, we took CN as a case study for EGBMMDA and prioritized the disease-related miRNAs (see Table 1). As a result, 9 of the top 10 and 43 of the top 50 potential CN-associated miRNAs were confirmed by experimental findings in dbDEMC and/or miR2Disease. In addition, six of the rest seven unconfirmed miRNAs were verified by more recent literatures than the databases. MiR-150 was reported to function as a key regulator in the tumorigenesis and progression of CN by targeting c-Myb^39^; miR-92a played a critical role in the CN development and an anti-miR-92a antagomir could lead to the apoptosis of CN cells^40^; miR-199a-3p, the 3p arm of the pre-miRNA for miR-199a, exhibited a higher expression in CN tissues, resulting a significantly lower survival rate for the patients^41^; miR-142-3p, the 3p arm of the pre-miRNA for miR-142, could suppress the CN cell growth via downregulating three CN-associated proteins CD133, Lgr5, and ABCG2^42^; an inverse correlation observed between the levels of miR-101 and the EP4 receptor protein in CN suggested that miR-101 might serve as a therapeutic target for the cancer^43^; miR-146b, with its expression inhibited, would lead to a high CsSR protein receptor level and reduce CN proliferation^44^. Consequently, 49 out of the top 50 potentially CN-related miRNAs were confirmed by either dbDEMC and miR2Disease or other experimental studies.

**Table 1:** Prediction of the top 50 predicted miRNAs associated with Colon Neoplasms based on known associations in HMDD database

miRNA	Evidence	miRNA	Evidence
hsa-mir-29a	dbDEMC;miR2Disease	hsa-let-7c	dbDEMC
hsa-mir-29b	dbDEMC;miR2Disease	hsa-mir-222	dbDEMC
hsa-let-7a	dbDEMC;miR2Disease	hsa-mir-199a	23292866
hsa-mir-143	dbDEMC;miR2Disease	hsa-mir-29c	dbDEMC
hsa-mir-150	25230975	hsa-mir-19a	dbDEMC;miR2Disease
hsa-mir-15a	dbDEMC	hsa-mir-142	23619912
hsa-mir-16	dbDEMC	hsa-mir-181a	dbDEMC;miR2Disease
hsa-mir-21	dbDEMC;miR2Disease	hsa-mir-125a	dbDEMC;miR2Disease
hsa-mir-1	dbDEMC;miR2Disease	hsa-mir-196a	dbDEMC;miR2Disease
hsa-mir-133a	dbDEMC;miR2Disease	hsa-mir-141	dbDEMC;miR2Disease
hsa-mir-146a	dbDEMC	hsa-mir-133b	dbDEMC;miR2Disease
hsa-mir-155	dbDEMC;miR2Disease	hsa-mir-10b	dbDEMC;miR2Disease
hsa-mir-200b	dbDEMC	hsa-mir-181b	dbDEMC;miR2Disease
hsa-mir-200c	dbDEMC;miR2Disease	hsa-mir-182	dbDEMC;miR2Disease
hsa-mir-20a	dbDEMC;miR2Disease	hsa-mir-183	dbDEMC;miR2Disease
hsa-mir-210	dbDEMC	hsa-mir-192	dbDEMC;miR2Disease
hsa-mir-221	dbDEMC;miR2Disease	hsa-mir-195	dbDEMC;miR2Disease
hsa-mir-223	dbDEMC;miR2Disease	hsa-mir-200a	Unconfirmed
hsa-mir-31	dbDEMC;miR2Disease	hsa-mir-203	dbDEMC;miR2Disease
hsa-mir-92a	21883694	hsa-mir-205	dbDEMC
hsa-mir-125b	dbDEMC	hsa-mir-34b	dbDEMC;miR2Disease
hsa-mir-18a	dbDEMC;miR2Disease	hsa-mir-93	dbDEMC;miR2Disease
hsa-mir-19b	dbDEMC;miR2Disease	hsa-let-7e	dbDEMC
hsa-mir-34a	dbDEMC;miR2Disease	hsa-mir-101	22353936
hsa-let-7b	dbDEMC;miR2Disease	hsa-mir-146b	26178670

The first column records top 1–25 related miRNAs. The third column records the top 26–50 related miRNAs. The evidences for the associations were either database studies or PMIDs of other experimental literatures

Lymphoma are mainly categorized into either Hodgkin lymphomas (HL) or non-Hodgkin lymphomas (NHL). In the United States in 2017, there are expected to be 8260 new HL patients and 72,240 NHL patients and a total number of 20,140 deaths^34^. An example of miRNAs associated with lymphoma is mir-19a, whose expression is upregulated in normal lymph nodes of canine B-cell lymphomas (a subtype of NHL)^45^. We took Lymphomas as the second case study and implemented EGBMMDA for predicting Lymphomas-related miRNAs. The results showed that 9 out of the top 10 potential miRNAs and 42 out of the top 50 potential miRNAs were confirmed by experimental literatures in dbDEMC and miR2Disease (see Table 2). In addition, three of the rest eight unverified miRNAs were verified by more recent literatures. Experimental data have shown that miR-193b experienced attenuation in cutaneous T-cell lymphoma^46^; by repressing miR-125b-5p (the 5p arm of the pre-miRNA for miR-125b), the Lymphoma cells would be sensitized to anticancer agents such as bortezomib^47^; the overexpression of miR-146b-5p (the 5p arm of the pre-miRNA for miR-146b) would prevent the cells of diffuse large B-cell lymphoma from growing^48^. Therefore, 45 out of the top 50 potentially lymphoma-related miRNAs were verified by either dbDEMC and miR2Disease or other experimental studies.

**Table 2:** Prediction of the top 50 predicted miRNAs associated with Lymphoma based on known associations in HMDD database

miRNA	Evidence	miRNA	Evidence
hsa-mir-196a	dbDEMC	hsa-mir-223	dbDEMC
hsa-mir-29a	dbDEMC	hsa-mir-25	dbDEMC
hsa-mir-29b	dbDEMC	hsa-mir-26b	dbDEMC
hsa-let-7a	dbDEMC	hsa-mir-31	dbDEMC
hsa-mir-141	dbDEMC	hsa-mir-34b	dbDEMC
hsa-mir-143	dbDEMC	hsa-mir-429	Unconfirmed
hsa-mir-145	dbDEMC	hsa-mir-93	dbDEMC
hsa-mir-1	dbDEMC	hsa-let-7e	dbDEMC
hsa-mir-133a	dbDEMC	hsa-mir-125b	23527180
hsa-mir-103a	Unconfirmed	hsa-mir-146b	24931464
hsa-mir-106a	dbDEMC	hsa-mir-148a	dbDEMC
hsa-mir-10b	dbDEMC	hsa-mir-196b	Unconfirmed
hsa-mir-151a	Unconfirmed	hsa-mir-219	dbDEMC
hsa-mir-152	dbDEMC	hsa-mir-27a	dbDEMC
hsa-mir-181b	dbDEMC	hsa-mir-27b	dbDEMC
hsa-mir-182	dbDEMC	hsa-mir-30a	dbDEMC
hsa-mir-183	dbDEMC	hsa-mir-30b	dbDEMC
hsa-mir-191	dbDEMC	hsa-mir-30c	dbDEMC
hsa-mir-192	dbDEMC	hsa-mir-338	dbDEMC
hsa-mir-193b	22235305	hsa-mir-34a	dbDEMC
hsa-mir-194	dbDEMC	hsa-mir-378a	Unconfirmed
hsa-mir-195	dbDEMC	hsa-mir-7	dbDEMC
hsa-mir-204	dbDEMC	hsa-mir-100	dbDEMC
hsa-mir-205	dbDEMC	hsa-mir-214	dbDEMC
hsa-mir-221	dbDEMC	hsa-mir-99a	dbDEMC

The first column records top 1–25 related miRNAs. The third column records the top 26–50 related miRNAs. The evidences for the associations were either database studies or PMIDs of other experimental literatures

PN is the second most common cancer diagnosed in males, with 161,360 new incidences and 26,730 deaths projected in the United States in 2017^34^. As indicated by studies^49–51^, miRNAs might complement existing PN detection methods as potential diagnostic biomarkers and promote the understanding of the cancer susceptibility at the genetic level. For instance, miR-221/222, miR-143/145, miR-23b/27b/24-1, and miR-1/133a experienced frequent downregulations in PN tissues and were viewed as tumor suppressors^51^. We took PN as the third case study and fitted EGBMMDA accordingly. Nine out of the top 10 and 45 out of the top 50 putative PN-associated miRNAs received biological verification by dbDEMC and miR2Disease (see Table 3). In addition, four of the rest five unsupported miRNAs were verified by more recent literatures. MiR-203 was indicated by a study^52^ as an anti-metastatic miRNA in PC, intervening the advancement of the cancer via repressing a cohort of premetastatic targets; miR-93 was commonly overexpressed in PC patients and worked collectively with miR-106b and miR-375 to attenuate Capicua levels and facilitate PC progression^53^; a reduction or loss of miR-146b expression was suggested as an omen of PC invasion by the literature^54^; miR-486-5p, the 5p arm of the pre-miRNA for miR-486, stagnated the migration and invasion of PC by lowering the protein expression of Snail, a key regulator of the epithelial–mesenchymal transition for cancer metastasis^55^. Provided these recent literature and database evidences, 49 out of the top 50 potentially PC-related miRNAs were verified.

**Table 3:** Prediction of the top 50 predicted miRNAs associated with Prostate Neoplasms based on known associations in HMDD database

miRNA	Evidence	miRNA	Evidence
hsa-mir-125a	dbDEMC;miR2Disease	hsa-mir-34c	dbDEMC
hsa-mir-196a	dbDEMC	hsa-mir-9	dbDEMC
hsa-mir-141	miR2Disease	hsa-mir-26a	dbDEMC;miR2Disease
hsa-mir-133b	dbDEMC	hsa-mir-206	dbDEMC
hsa-mir-181b	dbDEMC;miR2Disease	hsa-let-7f	dbDEMC;miR2Disease
hsa-mir-182	dbDEMC;miR2Disease	hsa-let-7g	dbDEMC;miR2Disease
hsa-mir-195	dbDEMC;miR2Disease	hsa-let-7i	dbDEMC
hsa-mir-200a	dbDEMC	hsa-mir-486	27877055
hsa-mir-203	21159887	hsa-mir-122	Unconfirmed
hsa-mir-205	dbDEMC;miR2Disease	hsa-mir-218	dbDEMC;miR2Disease
hsa-mir-34b	dbDEMC	hsa-mir-24	dbDEMC;miR2Disease
hsa-mir-93	26124181	hsa-mir-29a	dbDEMC;miR2Disease
hsa-let-7e	dbDEMC	hsa-mir-29b	dbDEMC;miR2Disease
hsa-mir-101	dbDEMC;miR2Disease	hsa-let-7a	dbDEMC;miR2Disease
hsa-mir-146b	21980038	hsa-mir-143	dbDEMC;miR2Disease
hsa-mir-148a	miR2Disease	hsa-mir-150	dbDEMC
hsa-mir-27a	dbDEMC;miR2Disease	hsa-mir-15a	dbDEMC;miR2Disease
hsa-mir-30a	miR2Disease	hsa-mir-16	dbDEMC;miR2Disease
hsa-mir-7	dbDEMC	hsa-mir-21	dbDEMC;miR2Disease
hsa-mir-100	dbDEMC;miR2Disease	hsa-mir-1	dbDEMC
hsa-mir-214	dbDEMC;miR2Disease	hsa-mir-133a	dbDEMC
hsa-let-7d	dbDEMC;miR2Disease	hsa-mir-146a	miR2Disease
hsa-mir-106b	dbDEMC	hsa-mir-155	dbDEMC
hsa-mir-15b	dbDEMC	hsa-mir-126	dbDEMC;miR2Disease
hsa-mir-124	dbDEMC	hsa-mir-17	miR2Disease

The first column records top 1–25 related miRNAs. The third column records the top 26–50 related miRNAs. The evidences for the associations were either database studies or PMIDs of other experimental literatures

Apart from predicting miRNAs for the three specific diseases, we also included in the supplementary materials a complete ranking list of potential miRNAs for all diseases in HMDD v2.0 (see Supplementary Table 1). The table consists of three columns: the disease’s name, the miRNA’s name, and their predicted association score.

To demonstrate the applicability of EGBMMDA to diseases having no known associated miRNAs, we carried out the fourth case study for Breast Neoplasms (BN) by removing all the known BN-related miRNAs in HMDD. This removal ensured that predicting candidate miRNAs for BN would only utilize the information of other diseases with known related miRNAs and the similarity information of diseases and miRNAs. There were 202 negated known BN-related miRNAs; and all 495 miRNAs in HMDD v2.0 were used as candidates. We ranked the candidates in terms of their predicted scores and validated the top 50 ones against HMDD v2.0 dbDEMC and miR2Disease. As a result, all 50 miRNAs were confirmed by these databases (see Table 4). Lastly, in the fifth case study, we assessed the performance of EGBMMDA trained by the older version of HMDD to see whether the model worked properly on a different dataset. This version of HMDD contained 1395 associations between 271 miRNAs and 137 diseases. Esophageal Neoplasms (EN) was chosen as the investigated disease. The predicted scores for candidate miRNAs were ranked and 49 out of the top 50 potentially EN-related miRNAs were confirmed by experimental findings recorded in dbDEMC, miR2Disease and HMDD v2.0 (see Table 5).

**Table 4:** Prediction of the top 50 predicted miRNAs associated with Breast Neoplasms based on known associations in HMDD database

miRNA	Evidence	miRNA	Evidence
hsa-mir-499a	HMDD	hsa-mir-132	dbDEMC;HMDD
hsa-mir-204	dbDEMC;miR2Disease;HMDD	hsa-mir-137	dbDEMC;HMDD
hsa-mir-26b	dbDEMC;HMDD	hsa-mir-206	dbDEMC;miR2Disease;HMDD
hsa-mir-95	dbDEMC	hsa-mir-23a	dbDEMC;HMDD
hsa-mir-219	dbDEMC;HMDD	hsa-mir-212	dbDEMC
hsa-mir-342	dbDEMC;HMDD	hsa-mir-125a	dbDEMC;miR2Disease;HMDD
hsa-mir-433	dbDEMC	hsa-let-7a	dbDEMC;miR2Disease;HMDD
hsa-mir-424	dbDEMC	hsa-mir-141	dbDEMC;miR2Disease;HMDD
hsa-mir-153	dbDEMC;HMDD	hsa-mir-143	dbDEMC;miR2Disease;HMDD
hsa-mir-181c	dbDEMC	hsa-mir-150	dbDEMC
hsa-mir-140	dbDEMC;HMDD	hsa-mir-133b	dbDEMC;HMDD
hsa-mir-328	dbDEMC;miR2Disease;HMDD	hsa-mir-106a	dbDEMC
hsa-mir-372	dbDEMC	hsa-mir-10b	dbDEMC;miR2Disease;HMDD
hsa-mir-373	dbDEMC;miR2Disease;HMDD	hsa-mir-126	dbDEMC;miR2Disease;HMDD
hsa-mir-708	HMDD	hsa-mir-181b	dbDEMC;miR2Disease;HMDD
hsa-mir-326	dbDEMC;HMDD	hsa-mir-182	dbDEMC;miR2Disease;HMDD
hsa-mir-302b	dbDEMC;HMDD	hsa-mir-183	dbDEMC;HMDD
hsa-mir-320a	HMDD	hsa-mir-192	dbDEMC
hsa-mir-506	HMDD	hsa-mir-195	dbDEMC;miR2Disease;HMDD
hsa-mir-516a	HMDD	hsa-mir-200a	dbDEMC;miR2Disease;HMDD
hsa-mir-184	dbDEMC	hsa-mir-200b	dbDEMC;miR2Disease;HMDD
hsa-mir-134	dbDEMC	hsa-mir-200c	dbDEMC;miR2Disease;HMDD
hsa-mir-32	dbDEMC	hsa-mir-203	dbDEMC;miR2Disease;HMDD
hsa-mir-325	dbDEMC	hsa-mir-205	dbDEMC;miR2Disease;HMDD
hsa-mir-30b	dbDEMC;HMDD	hsa-mir-223	dbDEMC;HMDD

The first column records top 1–25 related miRNAs. The third column records the top 26–50 related miRNAs

**Table 5:** Prediction of the top 50 predicted miRNAs associated with Esophageal Neoplasms based on known associations in the older version of the HMDD database

miRNA	Evidence	miRNA	Evidence
hsa-mir-20a	dbDEMC;HMDD	hsa-mir-34a	dbDEMC;HMDD
hsa-mir-221	dbDEMC	hsa-let-7c	dbDEMC;HMDD
hsa-mir-155	dbDEMC;HMDD	hsa-mir-29b	dbDEMC
hsa-mir-146a	dbDEMC;HMDD	hsa-mir-19b	dbDEMC
hsa-mir-222	dbDEMC	hsa-mir-126	dbDEMC;HMDD
hsa-mir-150	dbDEMC;HMDD	hsa-mir-206	dbDEMC
hsa-mir-1	dbDEMC	hsa-mir-9	dbDEMC
hsa-mir-143	dbDEMC;HMDD	hsa-mir-96	dbDEMC
hsa-mir-17	dbDEMC	hsa-mir-141	dbDEMC;HMDD
hsa-mir-125b	dbDEMC	hsa-mir-132	dbDEMC
hsa-mir-16	dbDEMC	hsa-mir-373	dbDEMC;miR2Disease
hsa-mir-133a	dbDEMC;HMDD	hsa-mir-451	dbDEMC
hsa-mir-181b	dbDEMC	hsa-mir-211	dbDEMC
hsa-mir-92a	HMDD	hsa-mir-142	dbDEMC
hsa-mir-15a	dbDEMC;HMDD	hsa-mir-494	dbDEMC
hsa-mir-18a	dbDEMC	hsa-mir-30c	dbDEMC
hsa-let-7d	dbDEMC	hsa-mir-302c	dbDEMC
hsa-mir-200b	dbDEMC	hsa-mir-10a	dbDEMC
hsa-mir-29a	dbDEMC	hsa-mir-34b	dbDEMC;HMDD
hsa-mir-19a	dbDEMC;HMDD	hsa-mir-377	dbDEMC
hsa-mir-145	dbDEMC;HMDD	hsa-mir-184	Unconfirmed
hsa-let-7b	dbDEMC;HMDD	hsa-mir-23b	dbDEMC
hsa-let-7a	dbDEMC;HMDD	hsa-mir-106b	dbDEMC
hsa-let-7e	dbDEMC	hsa-mir-199a	dbDEMC;HMDD
hsa-mir-223	dbDEMC;miR2Disease;HMDD	hsa-mir-196a	dbDEMC;miR2Disease;HMDD

The first column records top 1–25 related miRNAs. The third column records the top 26–50 related miRNAs

## Discussion

Identifying novel miRNA-disease associations promotes the understanding of disease pathogenesis from the perspective of miRNAs and benefits the treatment of diseases. In this study, we presented the computational model EGBMMDA under the hypothesis that functionally similar miRNAs are likely to be related to similar diseases. For biomedical researchers, identifying novel miRNA–disease associations enhances their understanding towards the molecular mechanisms of diseases at the miRNA level and benefits the development of disease diagnostic biomarkers and therapeutic tools. Our model could be a valuable complement to experimental methods for discovering miRNA–disease connections: researchers could use EGBMMDA to computationally infer the miRNAs that were potentially associated with the disease of interest, then rank these miRNAs by association scores, and finally choose the most promising associations for biological confirmation. In this manner, experiments could be more effective and productive. The informative feature vector *I* for the miRNA–disease pair (*m*(*i*),*d*(*j*)) was constructed via feature extraction on the miRNA functional similarity, the disease semantic similarity, and the known miRNA–disease associations, and was fed into the model for prediction. The result was the association score for this pair. The higher the score for miRNA *m*(*i*) and disease *d*(*j*) was, the more likely *m*(*i*) was associated with *d*(*j*). Desirable evaluation outcomes were obtained from both cross-validations (LOOCV and fivefold) and case studies on CN, Lymphoma, PN, BN, and EN. EGBMMDA outperformed eight earlier models MiRAI, MCMDA, HGIMDA, MIDP, WBSMDA, RLSMDA, HDMP, and RWRMDA. We believe that it is the first decision tree learning-based computational model applied to predicting potential miRNA–disease associations.

Three factors contributed to the reliable performance of EGBMMDA. First, heterogeneous datasets including the miRNA functional similarity, the disease semantic similarity, and known miRNA–disease associations were merged into a feature vector *I* for learning the model. The vector *I* included the statistical measures (such as sum, mean, histogram distributions of similarity scores), the graph theory-related measures (such as neighbor count, betweenness, closeness and eigenvector centrality, and Page-Rank scores of miRNA/disease adjacency matrices), and matrix factorization of the miRNA–disease association network. Consequently, EGBMMDA took the advantage of the exhaustive information about each miRNA–disease pair. Second, the model was based upon a scalable tree boosting system^56^. While in this study EGBMMDA was fitted by thousands of instances with more than a hundred feature dimensions, it actually had the potential of dealing with even larger datasets. Third, the tree boosting system was fundamentally an ensemble machine learning algorithm where each split made during the tree growth was an optimal operation that combined with all other splits to minimize the total loss function. Therefore, the finished tree was able to make accurate predictions.

Nevertheless, limitations exist in the model. Unlike another machine learning-based RLSMDA model, EGBMMDA required its training data to have both positive and negative samples. To resolve this issue, we had randomly selected a subset of unknown miRNA–disease associations as negative instances. Though the fivefold cross-validation results indicated EGBMMDA to be a relatively stable model with a 0.0012 standard deviation of AUCs, to what extent incorrectly chosen negative samples would affect the model’s prediction accuracy deserves further investigation. Moreover, more reliably calculated disease similarity and miRNA similarity could improve the performance of the model. We expect more biologically relevant information to be available in the future to refine the similarity measures. In addition, more experimentally confirmed miRNA–disease associations would help eliminate the bias of the learning algorithm for EGBMMDA. Moreover, our current analysis did not include the tissue specific expression of miRNAs, so it was difficult to examine how much of our model’s prediction ability was attributed to the abundance of miRNA and mRNA in the respective tissue. We would consider this issue in future research. Lastly, the three databases used in this study had variable quality because they were created at various times, under different methodologies and from diverse data sources. We expect newer and more comprehensive databases to be released in the future, so that both evaluating computational models and predicting novel miRNA–disease associations would become more reliable.

## Materials and methods

### LOOCV and fivefold cross-validation

To evaluate the prediction accuracy of EGBMMDA, we implemented global and local LOOCV frameworks. Using cross-validations as the evaluation scheme for computational models is the standard practice in the field of miRNA–disease association prediction. This scheme has been adopted in many previous studies^21–25,27,31^. In global LOOCV, each known miRNA–disease association was left out in turn as test association. All the other known associations were regarded as seeds, while those miRNA–disease pairs without any evidence (including the left-out pair) to prove their associations were considered as candidates. It is worth mentioning that throughout the cross-validations and case studies in performance evaluation, each time of fitting an EGBMMDA model, seeds were used as positive training samples and an equal number of samples were randomly selected as negative training examples from the pool of unknown associations. This operation guaranteed a balanced training dataset with half positive and half negative instances. The predicted score for the test association was ranked relative to the scores for candidates and, if its ranking was above a given threshold, we obtained a successful prediction made by the model. Local LOOCV, in contrast, focused on rankings of miRNAs for a specific disease. For the disease *d*(*i*), each known miRNA related to it was left out in turn as the test miRNA. All the other known disease-related miRNAs (including ones for diseases other than disease *d*(*i*)) were regarded as seeds, whereas those without any evidence to confirm their associations with disease *d*(*i*) (including the left-out miRNA) were considered as candidates. The predicted score for the test miRNA *m*(*j*) was ranked relative to the scores for candidate miRNAs; and if the ranking exceeded a given threshold, the model was rendered to correctly predict the *m*(*j*)–*d*(*i*) association. In short, the difference between global and local LOOCV was whether all diseases were considered simultaneously in the ranking or not. Although we did not set the threshold score for a positive association prediction in our study, various ranking thresholds were applied in cross-validations. We ranked the test sample and candidates in terms of their association scores. The test sample would be a positive prediction if it was ranked above a threshold, and a negative prediction otherwise. The true positive rate (TPR, sensitivity) and the true negative rate (FPR, 1-specificity) were calculated corresponding to each ranking threshold, so that enough points would be obtained to plot the ROC curve. Sensitivity denotes the proportion of test samples whose rankings are higher than the threshold, whereas specificity means the percentage of candidates whose rankings are lower than the threshold. From the ROC curve, we calculated the evaluation metric AUC.

To further evaluate the stability of EGBMMDA, we implemented fivefold cross-validation where the known miRNA–disease associations were randomly partitioned into five equally-sized subsets. Four subsets were regarded as training samples to learn the model and the other subset was used as the test samples. Similar to the case of global LOOCV, the known miRNA–disease associations were seeds and the miRNA–disease pairs without known association evidences were candidates. The predicted scores of the test samples were ranked against the scores of candidates. The fivefold CV procedure was randomly repeated for 100 times to acquire a more accurate estimate of the EGBMMDA prediction performance.

### Human miRNA–disease associations

The human miRNA–disease association dataset used to train EGBMMDA was retrieved from HMDD v2.0^14^, covering 5430 experimentally confirmed associations between 495 miRNAs and 383 diseases (see Supplementary Table 2). Variables *nm* and *nd* denoted the number of miRNAs and diseases, respectively; and an *nm* × *nd* adjacency matrix (a network graph made up of miRNAs and diseases as vertices) was established to better represent miRNA–disease associations. An entity *A*(*m*(*i*),*d*(*j*)) equaled 1 if miRNA *m*(*i*) had a verified connection to disease *d*(*j*) and 0 otherwise.

### MiRNA functional similarity

MiRNA functional similarity scores were calculated under the assumption that functionally similar miRNAs are more likely to connect with phenotypically similar diseases^57^. We downloaded the scores from http://www.cuilab.cn/files/images/cuilab/misim.zip and constructed an *nm* × *nm* miRNA functional similarity matrix *FS* where an entity *FS*(*m*(*i*),*m*(*j*)) represented the similarity score between miRNA *m*(*i*) and *m*(*j*).

### Disease semantic similarity

Disease semantic similarity scores were computed according to the methodology adopted in he literature^22^. A disease can be described by a Directed Acyclic Graph (DAG) in which the nodes represent the disease and its ancestor diseases and a directed edge from a parent node to a child node represents the relationship between the two nodes. The contribution of disease *t* in DAG(*d*(*i*)) to the semantic value of disease *d*(*i*) was defined by

1 $$D_{d\left( i \right)}\left( t \right) = - {\mathrm{log}}\left( {\frac{{{\mathrm{the}}\,{\mathrm{number}}\,{\mathrm{of}}\,{\mathrm{DAGs}}\,{\mathrm{including}}\,t}}{{{\mathrm{the}}\,{\mathrm{number}}\,{\mathrm{of}}\,{\mathrm{diseases}}}}} \right)$$

which meant that a more specific disease *t* should make a greater contribution to the semantic value of the investigated disease *d*(*i*). The semantic value of *d*(*i*) was given by the summation of all the contributions from ancestor diseases and disease *d*(*i*) itself

2 $$DV\left( {d\left( i \right)} \right) = \mathop {\sum }\limits_{t \in D\left( {d\left( i \right)} \right)} D_{d\left( i \right)}\left( t \right)$$

where *D*(*d*(*i*)) was the node set in DAG*d*(*i*) including node *d*(*i*) itself. It should be obvious that two diseases sharing larger part of their DAGs tended to have a higher semantic similarity score. Therefore, the semantic similarity between disease *d*(*i*) and *d*(*j*) could be defined as follows:

3 $$SS\left( {d\left( i \right),d\left( j \right)} \right) = \frac{{\mathop {\sum }\nolimits_{t \in D\left( {d\left( i \right)} \right) \cap D\left( {d\left( j \right)} \right)} \left( {D_{d\left( i \right)}\left( t \right) + D_{d\left( j \right)}\left( t \right)} \right)}}{{DV\left( {d\left( i \right)} \right) + DV\left( {d\left( j \right)} \right)}}$$

where *SS* was an *nd* × *nd* disease semantic similarity matrix.

### EGBMMDA

**Fig. 2:**
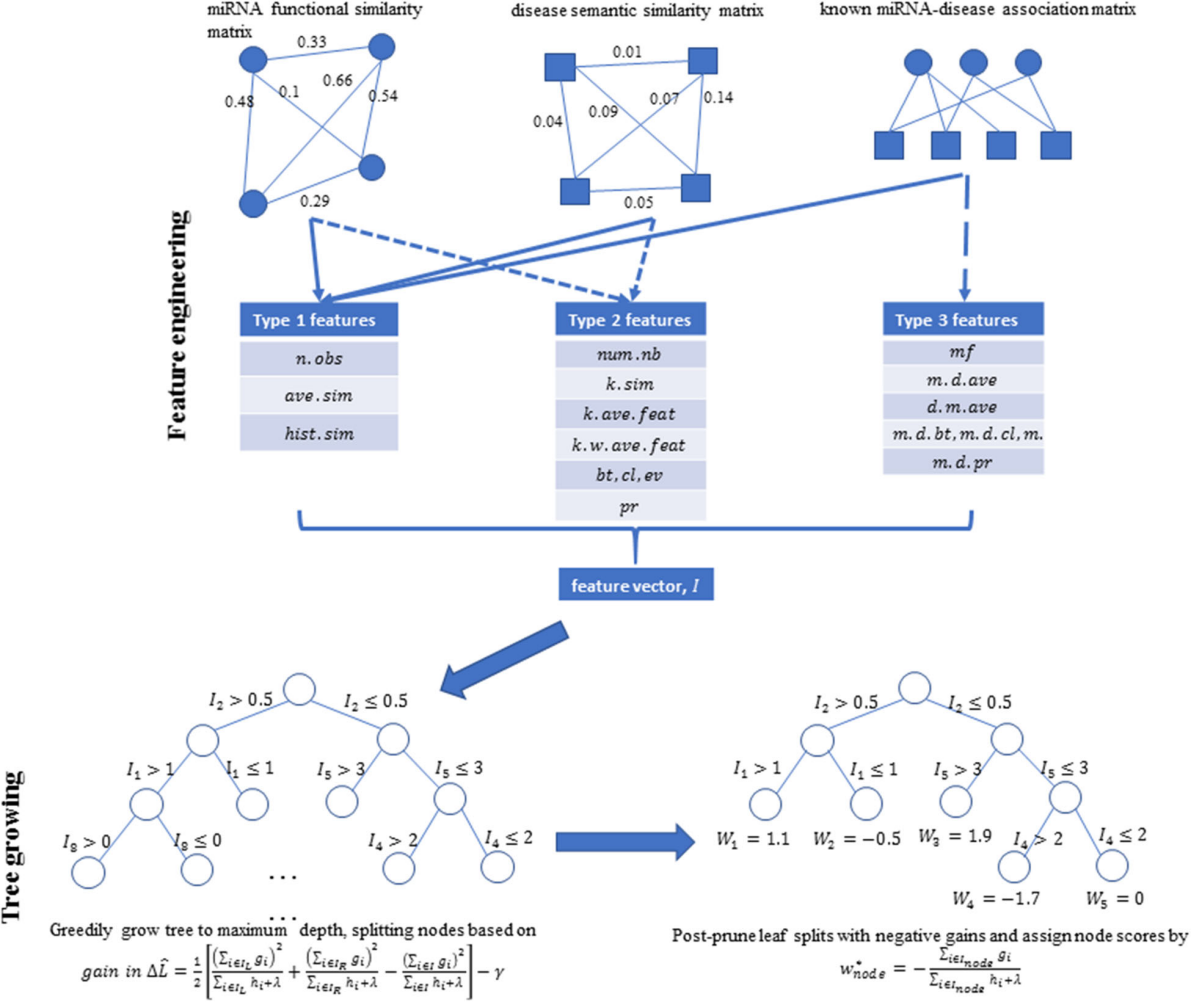
Flowchart of potential miRNA–disease association prediction based on the computational model of EGBMMDA

The EGBMMDA model was implemented by integrating the miRNA–disease association matrix *A*, the miRNA functional similarity matrix *FS* and the disease semantic similarity matrix *SS*. Specifically, the implementation involved two steps as depicted in Fig. 2: the feature engineering step where the three matrices were merged into a feature vector *I* and the regression tree growing step where a regression tree was grown based on *I* and under the gradient boosting framework. The scripts for the complete implementation of EGBMMDA are available at http://www.escience.cn/system/file?fileId=91170.

There were three types of vectors constructed during feature engineering (see Table 6), similar to those introduced by the literature^58^. Type 1 features included the statistical measures summarized for each individual miRNA/disease in *A*, *FS*, and *SS*. For the miRNA *m*(*i*)/disease *d*(*j*), *n*.*obs* denoted the number of observed associations in the corresponding *i*th row/*j*th column of *A*; *ave*.*sim* denoted the average of all similarity scores, namely, the average of the *i*th/*j*th row of *FS*/*SS*; *hist*.*sim* denoted the histogram feature where the range of similarity scores [0, 1] was segmented into *n* bins (*n* = 5 in this study) and we counted the proportion of similarity scores for *m*(*i*)/*d*(*j*) that fell into each bin.

**Table 6:** Feature vector extracted from the miRNA functional similarity matrix, the disease semantic similarity matrix, and the known miRNA–disease association matrix

Type 1 features for each miRNA/disease	*n*.*obs*	For the miRNA *m*(*i*)/disease *d*(*j*), the number of observed associations in the corresponding *i*th row/*j*th column of *A*
	*ave*.*sim*	The average of all similarity scores, namely, the average of the *i*th/*j*th row of *FS*/*SS*
	*hist*.*sim*	The range of similarity scores [0, 1] was segmented into *n* bins and we counted the proportion of similarity scores for *m*(*i*)/*d*(*j*) that fell into each bin
Type 2 features for each miRNA/disease	*num*.*nb*	Number of neighbors of a node in the unweighted graph version of *FS*/*SS*
	*k*.*sim*	The similarity values of the k-nearest neighbors of a node
	*k*.*ave.feat*	The average of Type 1 features among the k-nearest neighbors of a node
	*k.w*.*ave.feat*	The average of Type 1 features among the k-nearest neighbors of a node weighted by the similarity values.
	*bt*,*cl*,*ev*	Betweenness, closeness, eigenvector centrality of a node
	*pr*	Page-Rank score of a node
Type 3 features for each miRNA–disease pair	*mf*	Latent vectors for the miRNA and the disease, obtained by matrix factorization of *A*
	*m*.*d.ave*	The number of associations between an miRNA and a disease’s neighbors
	*d*.*m.ave*	The number of associations between a disease and an miRNA’s neighbors
	*m*.*d*.*bt,m.d.cl,m.d.ev*	Betweenness, closeness, eigenvector centrality of a node
	*m*.*d.pr*	Page-Rank score of a node

Type 2 features covered graph theory-related statistics for nodes in *FS*/*SS*. An edge between two nodes existed if their similarity score exceeded the mean value of all entities in *FS*/*SS*. In this way, we built the unweighted graph version of *FS*/*SS*, and from which we extracted with respect to each node: (1) *num*.*nb*, the number of its neighbors; (2) *k*.*sim*, similarity values of its k-nearest neighbors (*k* = 10 in this study); (3) *k*.*ave.feat*, its average of Type 1 features among the k-nearest neighbors; (4) *k*.*w*.*ave.feat*, its average of Type 1 features among the k-nearest neighbors weighted by the similarity values; (5) *bt*,*cl*,*ev*, its respective betweenness, closeness, and eigenvector centrality; (6) *pr*, its Page-Rank score.

Type 3 features focused on each miRNA-disease pair (*m*(*i*),*d*(*j*)) in the association matrix *A*. We carried out matrix factorization (*mf*) of *A* and recorded the latent vectors for *m*(*i*) and *d*(*j*). In addition, we further included the number of associations between *m*(*i*) and *d*(*j*)’s neighbors (denoted by *m*.*d*.*ave*) and the number of associations between *d*(*j*) and *m*(*i*)’s neighbors (denoted by *d*.*m*.*ave*). Furthermore, the betweenness *m*.*d*.*bt*, closeness *m*.*d*.*cl*, and eigenvector *m*.*d*.*ev* centralities and Page-Rank scores *m*.*d*.*pr* for *m*(*i*) and *d*(*j*) were also calculated to make full use of *A*.

A composite feature vector was produced by concatenating these three feature types and used to train EGBMMDA. The feature vector for the (*m*(*i*),*d*(*j*)) pair had the general form of

4 $$feature\,vector\,{\mathrm{for}}\,\left( {m\left( i \right),d\left( j \right)} \right) = \left[ {\begin{array}{*{20}{c}} {Type\,1\,of\,m\left( i \right)} \\ {Type\,1\,of\,d\left( j \right)} \\ {Type\,2\,of\,m\left( i \right)} \\ {Type\,2\,of\,d\left( j \right)} \\ {Type\,3\,of\,\left( {m\left( i \right),d\left( j \right)} \right)} \end{array}} \right]$$

**Fig. 3:**
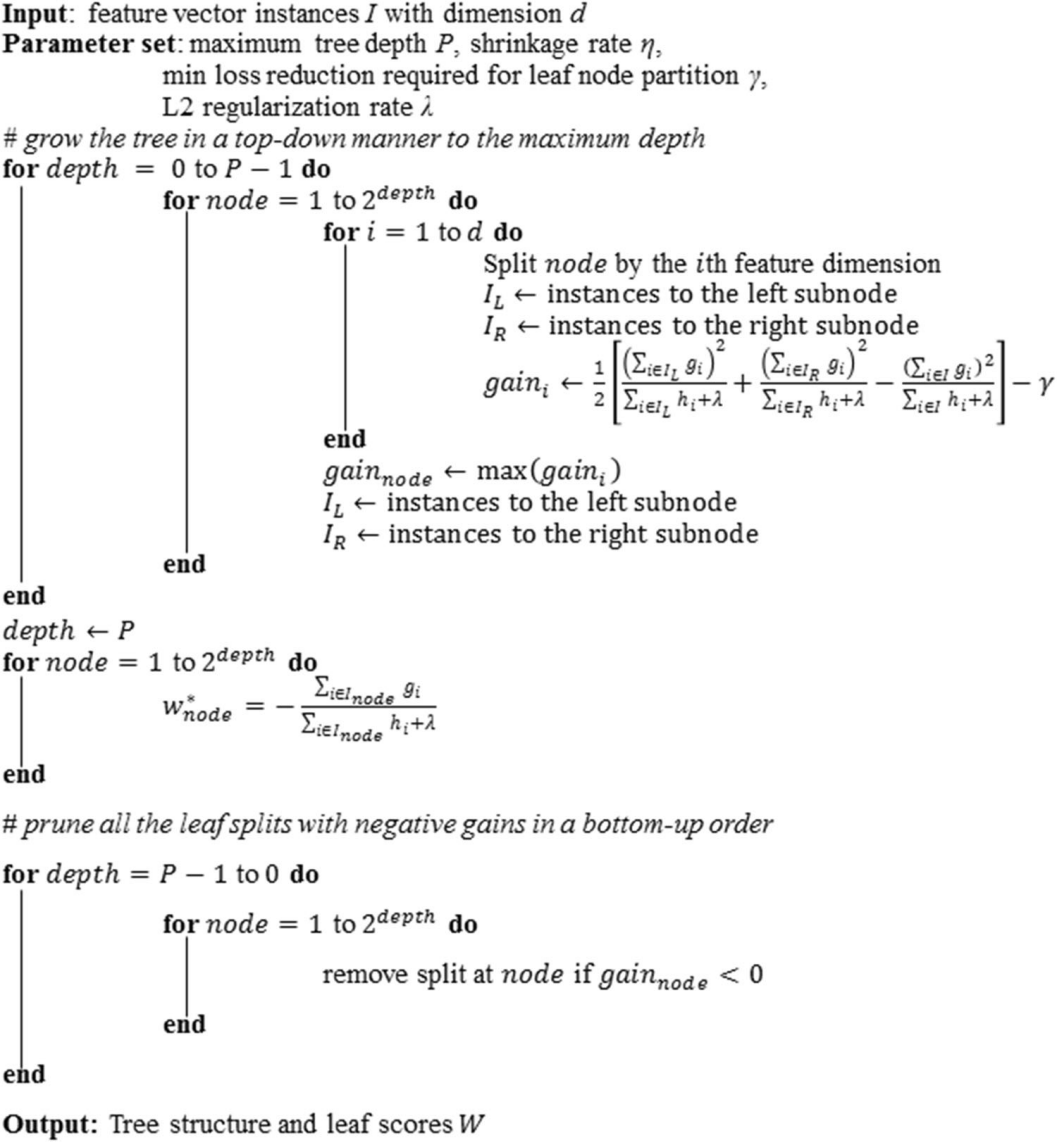
Tree growing algorithm. The algorithm first grew the tree in a top-down manner to the maximum depth specified by the user, creating a 2^depth^ number of nodes, and then pruned all the leaves with negative gains in a bottom-up order

EGBMMDA grew the regression tree by following a greedy-growth-and-post-pruning process. The model took the feature vector *I* as input and output the tree splits based on *I* and the corresponding leaf scores *W*. The parameter set included the maximum tree depth *P*, the shrinkage rate *η*, the minimum loss reduction required to partition a leaf node of the tree *γ*, and the L2 regularization rate *λ*. Throughout this study, we used *P* = 6, *γ* = 0, *λ* = 1 based on the default parameter set of the extreme gradient boosting training package implemented according to Chen et al.^56^. The package is available at https://github.com/dmlc/xgboost. In addition, we used *η* = 1 to impose no step-size shrinkage on the boosting process, as with the literature^58^. All the parameters could be optimized via cross-validation. The algorithm first grew the tree in a top-down manner to the maximum depth *P* specified by the user, creating a 2^*P*^ number of nodes, and then pruned all the leaf splits with negative gains in a bottom-up order (see Fig. 3). The criterion for splitting a leaf node was based on a *gain in loss reduction* equation. According to the literatures^56,58,59^, the derivation of the equation is illustrated as follows.

EGBMMDA was an ensemble model where regression trees were used as functions in a gradient boosting framework, which trained a sequence of weak learners *f*_*k*_ to collectively make a predicted score $\hat y_i$ in a functional form like this

5 $$\hat y_i = \mathop {\sum }\limits_{k = 1}^K f_k\left( {{\boldsymbol{x}}_{\boldsymbol{i}}} \right),f_k \in F$$

where ***x***_*i*_ was the input vector, *K* was the number of regression functions, and *F* was the space of all possible *f*_*k*_s. The objective function for learning the set of *f*_*k*_s was given by

6 $$\mathop {{\min }}\limits_{f_k} \mathop {\sum }\limits_{i = 1}^n l\left( {y_i,\hat y_i} \right) + \mathop {\sum }\limits_{k = 1}^K {\mathrm{\Omega }}\left( {f_k} \right)$$

where *l* was a loss function between the observed value *y*_*i*_ and predicted value $\hat y_i$ and Ω in the regularization term penalized the model complexity to avoid overfitting. The model was trained iteratively and additively: at the *t*th iteration, a new function *f*_*t*_ selected from *F* was added to the ensemble to predict $\hat y_i^{\left( t \right)}$. $\hat y_i^{\left( t \right)}$ was the prediction for the *i*th instance at the *t*th iteration. The selection of *f*_*t*_ should optimize the *t*th objective function as

7 $$\mathop {{\min }}\limits_{f_t} \mathop {\sum }\limits_{i = 1}^n l\left( {y_i,\hat y_i^{\left( t \right)}} \right) + \mathop {\sum }\limits_{j = 1}^t {\mathrm{\Omega }}\left( {f_j} \right)$$

which could alternatively be rewritten as

8 $$\mathop {{\min }}\limits_{f_t} \mathop {\sum }\limits_{i = 1}^n l\left( {y_i,\hat y_i^{\left( {t - 1} \right)} + f_t\left( {{\boldsymbol{x}}_{\boldsymbol{i}}} \right)} \right) + \mathop {\sum }\limits_{j = 1}^t {\mathrm{\Omega }}\left( {f_j} \right)$$

Usually the addition of *f*_*t*_ was multiplied by a shrinkage parameter *η* to avoid overfitting. To simplify the optimization of (9), the loss function *l* was expanded according to the second-order Taylor series $f\left( x \right) = f\left( a \right) + \frac{{f^{'}\left( a \right)}}{{1!}}\left( {x - a} \right) + \frac{{f^{{''}}\left( a \right)}}{{2!}}\left( {x - a} \right)^2$. Let *x* be $\hat y_i^{\left( {t - 1} \right)} + f_t\left( {{\boldsymbol{x}}_{\boldsymbol{i}}} \right)$ and *a* be $\hat y_i^{\left( {t - 1} \right)}$, the objective function reduced to

9 $$\mathop {{\min }}\limits_{f_t} \mathop {\sum }\limits_{i = 1}^n \left[ {l\left( {y_i,\hat y_i^{\left( {t - 1} \right)}} \right) + g_if_t\left( {{\boldsymbol{x}}_{\boldsymbol{i}}} \right) + \frac{1}{2}h_if_t^2\left( {{\boldsymbol{x}}_{\boldsymbol{i}}} \right)} \right] + \mathop {\sum }\limits_{j = 1}^t {\mathrm{\Omega }}\left( {f_j} \right)$$

where $g_i = \delta _{\hat y_i^{\left( {t - 1} \right)}}l\left( {y_i,\hat y_i^{\left( {t - 1} \right)}} \right)$ was the first derivative of *l* and $h_i = \delta _{\hat y_i^{\left( {t - 1} \right)}}^2l\left( {y_i,\hat y_i^{\left( {t - 1} \right)}} \right)$ was the second derivative of *l*. Removing the constant terms in (10) gave

10 $$\mathop {{\min }}\limits_{f_t} \mathop {\sum }\limits_{i = 1}^n \left[ {g_if_t\left( {{\boldsymbol{x}}_{\boldsymbol{i}}} \right) + \frac{1}{2}h_if_t^2\left( {{\boldsymbol{x}}_{\boldsymbol{i}}} \right)} \right] + {\mathrm{\Omega }}\left( {f_t} \right)$$

The algorithm iteratively added a function *f*_*t*_ that optimized (10) for each iteration. In EGBMMDA, *f*_*t*_ was given by a series of discrete functions. The feature vectors ***x***_*i*_ were divided into *T* regions and each region was assigned an independent weight. The mapping of ***x***_*i*_ to the indices of the regions was defined by $q:R^d \to \left( {1,2,3, \ldots ,T} \right)$ and the vector ***w*** denoted the weight for each region. Therefore, *f*_*t*_ should be

11 $$f_t\left( {\mathbf{x}} \right) = w_{q\left( {\boldsymbol{x}} \right)}$$

Moreover, the regularization term of (11) was defined by

12 $${\mathrm{\Omega }}\left( {f_t} \right) = \gamma T + \frac{1}{2}\lambda \mathop {\sum }\limits_{j = 1}^T w_j^2$$

where *γ* and *λ* were the trade-off parameters. Equation (13) penalized both the number of regions *T* and the sum of squared weight $w_j^2$ for each region to avoid overfitting. The implication of (12) and (13) was that the algorithm would search for the optimal segmentation structure *q* and weight vector ***w***. This corresponded to the optimization over the tree structure and node scores when growing the regression tree. By denoting the instance set in Region *j* as $I_j = \left( {i|q\left( {{\boldsymbol{x}}_{\boldsymbol{i}}} \right) = j} \right)$ and substituting (12) and (13) into (11), the objective function for each iteration became

13 $$\mathop {{\min }}\limits_{f_t} \mathop {\sum}\limits_{i = 1}^n {\left[ {g_if_t\left( {{\boldsymbol{x}}_{\boldsymbol{i}}} \right) + \frac{1}{2}h_if_t^2\left( {{\boldsymbol{x}}_{\boldsymbol{i}}} \right)} \right] + \gamma T + \frac{1}{2}\lambda \mathop {\sum }\limits_{j = 1}^T w_j^2} \\  = \mathop {{\min }}\limits_{w_j} \mathop {\sum }\limits_{j = 1}^T \left[ {\left( {\mathop {\sum }\limits_{i \in I_j} g_i} \right)w_j + \frac{1}{2}\left( {\mathop {\sum }\limits_{i \in I_j} h_i + \lambda } \right)w_j^2} \right] + \gamma T$$

Taking the derivatives of (14) with respect to *w*_*j*_ and equating them to zero gave the optimal weight $w_j^{\mathrm{*}}$ of region *j*

14 $$w_j^{\mathrm{*}} = - \frac{{\mathop {\sum }\nolimits_{i \in I_j} g_i}}{{\mathop {\sum }\nolimits_{i \in I_j} h_i + \lambda }}$$

The optimal objective function value could be obtained by plugging (15) back into (14)

15 $$\hat L^{\left( t \right)}\left( q \right) = - \frac{1}{2}\mathop {\sum }\limits_{j = 1}^T \frac{{\left( {\mathop {\sum }\nolimits_{i \in I_j} g_i} \right)^2}}{{\mathop {\sum }\nolimits_{i \in I_j} h_i + \lambda }} + \gamma T$$

We used *I*_L_ and *I*_R_ $(I_{\mathrm{L}} \cup I_{\mathrm{R}} = I)$ to denote the instance sets of left and right sub-nodes of a node split. The gain in loss reduction of (15) resulted from the split was hence

16 $${\mathrm{gain}}\,{\mathrm{in}}\,\Delta \hat L = \frac{1}{2}\left[ {\frac{{\left( {\mathop {\sum }\nolimits_{i \in I_L} g_i} \right)^2}}{{\mathop {\sum }\nolimits_{i \in I_L} h_i + \lambda }} + \frac{{\left( {\mathop {\sum }\nolimits_{i \in I_R} g_i} \right)^2}}{{\mathop {\sum }\nolimits_{i \in I_R} h_i + \lambda }} - \frac{{\left( {\mathop {\sum }\nolimits_{i \in I} g_i} \right)^2}}{{\mathop {\sum }\nolimits_{i \in I} h_i + \lambda }}} \right] - \gamma$$

which was the *gain in loss reduction* equation and utilized as the criterion for splitting leaf nodes during the tree growth.

## Electronic supplementary material

Supplementary material

Supplementary Table 1

Supplementary Table 2

## References

[1] Pfeffer S (2005). Identification of microRNAs of the herpesvirus family. Nat. Methods.

[2] Reinhart BJ, Weinstein EG, Rhoades MW, Bartel B, Bartel DP (2002). MicroRNAs in plants. Genes Dev..

[3] Rodriguez A, Griffiths-Jones S, Ashurst JL, Bradley A (2004). Identification of mammalian microRNA host genes and transcription units. Genome Res..

[4] Ambros V (2004). The functions of animal microRNAs. Nature.

[5] Bartel DP (2009). MicroRNAs: target recognition and regulatory functions. Cell.

[6] Miska EA (2005). How microRNAs control cell division, differentiation and death. Curr. Opin. Genet. Dev..

[7] Karp X, Ambros V (2005). Developmental biology. Encountering microRNAs in cell fate signaling. Science.

[8] Zhen L, Sall A, Yang D (2008). MicroRNA: an Emerging Therapeutic Target and Intervention Tool. Int. J. Mol. Sci..

[9] Gregory RI, Shiekhattar R (2005). MicroRNA biogenesis and cancer. Cancer Res..

[10] Yu Z (2007). Aberrant allele frequencies of the SNPs located in microRNA target sites are potentially associated with human cancers. Nucleic Acids Res..

[11] Calin GA (2002). Frequent deletions and down-regulation of micro-RNA genes miR15 and miR16 at 13q14 in chronic lymphocytic leukemia. Proc. Natl Acad. Sci. USA.

[12] Cai J (2012). MicroRNA-200 is commonly repressed in conjunctival MALT lymphoma, and targets cyclin E2. Graefes Arch. Clin. Exp. Ophthalmol..

[13] Ueno K (2013). microRNA-183 is an oncogene targeting Dkk-3 and SMAD4 in prostate cancer. Br. J. Cancer.

[14] Li Y (2014). HMDDv2.0: a database for experimentally supported human microRNA and disease associations. Nucleic Acids Res..

[15] Yang Z (2010). dbDEMC: a database of differentially expressed miRNAs in human cancers. BMC Genomics.

[16] Jiang Q (2009). miR2Disease: a manually curated database for microRNA deregulation in human disease. Nucleic Acids Res..

[17] Calin GA, Croce CM (2006). MicroRNA signatures in human cancers. Nat. Rev. Cancer.

[18] Perez-Iratxeta C, Bork P, Andrade MA (2002). Association of genes to genetically inherited diseases using data mining. Nat. Genet..

[19] Perez-Iratxeta C, Wjst M, Bork P, Andrade MA (2005). G2D: a tool for mining genes associated with disease. BMC Genet..

[20] Aerts S (2006). Gene prioritization through genomic data fusion. Nat. Biotechnol..

[21] Jiang Q (2010). Prioritization of disease microRNAs through a human phenome-microRNAome network. BMC Syst. Biol..

[22] Xuan P (2013). Prediction of microRNAs associated with human diseases based on weighted k most similar neighbors. PLoS ONE.

[23] Chen X, Liu MX, Yan GY (2012). RWRMDA: predicting novel human microRNA-disease associations. Mol. Biosyst..

[24] Xuan P (2015). Prediction of potential disease-associated microRNAs based on random walk. Bioinformatics.

[25] Chen X (2016). WBSMDA: within and between score for MiRNA-disease association prediction. Sci. Rep..

[26] Chen X (2016). HGIMDA: heterogeneous graph inference for miRNA-disease association prediction. Oncotarget.

[27] Li JQ, Rong ZH, Chen X, Yan GY, You ZH (2017). MCMDA: Matrix Completion for MiRNA-Disease Association prediction. Oncotarget.

[28] Shi H (2013). Walking the interactome to identify human miRNA-disease associations through the functional link between miRNA targets and disease genes. BMC Syst. Biol..

[29] Mork S, Pletscher-Frankild S, Palleja Caro A, Gorodkin J, Jensen LJ (2014). Protein-driven inference of miRNA-disease associations. Bioinformatics.

[30] Pasquier C, Gardes J (2016). Prediction of miRNA-disease associations with a vector space model. Sci. Rep..

[31] Xu J (2011). Prioritizing candidate disease miRNAs by topological features in the miRNA target-dysregulated network: case study of prostate cancer. Mol. Cancer Ther..

[32] Chen X, Yan GY (2014). Semi-supervised learning for potential human microRNA-disease associations inference. Sci. Rep..

[33] Chen X (2015). RBMMMDA: predicting multiple types of disease-microRNA associations. Sci. Rep..

[34] Siegel RL, Miller KD, Jemal A (2017). Cancer statistics, 2017. CA Cancer J. Clin..

[35] Ogata-Kawata H (2014). Circulating exosomal microRNAs as biomarkers of colon cancer. PLoS ONE.

[36] Guo C (2008). The noncoding RNA, miR-126, suppresses the growth of neoplastic cells by targeting phosphatidylinositol 3-kinase signaling and is frequently lost in colon cancers. Genes Chromosomes Cancer.

[37] Shi B (2007). Micro RNA 145 targets the insulin receptor substrate-1 and inhibits the growth of colon cancer cells. J. Biol. Chem..

[38] Drusco A (2014). MicroRNA profiles discriminate among colon cancer metastasis. PLoS ONE.

[39] Feng J (2014). miR-150 functions as a tumour suppressor in human colorectal cancer by targeting c-Myb. J. Cell. Mol. Med..

[40] Tsuchida A (2011). miR-92 is a key oncogenic component of the miR-17-92 cluster in colon cancer. Cancer Sci..

[41] Wan D (2013). Aberrant expression of miR-199a-3p and its clinical significance in colorectal cancers. Med. Oncol..

[42] Shen WW, Zeng Z, Zhu WX, Fu GH (2013). MiR-142-3p functions as a tumor suppressor by targeting CD133, ABCG2, and Lgr5 in colon cancer cells. J. Mol. Med. (Berl.).

[43] Chandramouli A (2012). MicroRNA-101 (miR-101) post-transcriptionally regulates the expression of EP4 receptor in colon cancers. Cancer Biol. Ther..

[44] Fetahu IS (2016). miR-135b- and miR-146b-dependent silencing of calcium-sensing receptor expression in colorectal tumors. Int. J. Cancer.

[45] Uhl E, Krimer P, Schliekelman P, Tompkins SM, Suter S (2011). Identification of altered MicroRNA expression in canine lymphoid cell lines and cases of B- and T-cell lymphomas. Genes Chromosomes Cancer.

[46] Manfe V (2012). miR-122 regulates p53/Akt signalling and the chemotherapy-induced apoptosis in cutaneous T-cell lymphoma. PLoS ONE.

[47] Manfe V (2013). cMyc/miR-125b-5p signalling determines sensitivity to bortezomib in preclinical model of cutaneous T-cell lymphomas. PLoS ONE.

[48] Wu PY, Zhang XD, Zhu J, Guo XY, Wang JF (2014). Low expression of microRNA-146b-5p and microRNA-320d predicts poor outcome of large B-cell lymphoma treated with cyclophosphamide, doxorubicin, vincristine, and prednisone. Hum. Pathol..

[49] Zhang W (2014). Identification of candidate miRNA biomarkers from miRNA regulatory network with application to prostate cancer. J. Transl. Med..

[50] Watahiki A (2013). Plasma miRNAs as biomarkers to identify patients with castration-resistant metastatic prostate cancer. Int. J. Mol. Sci..

[51] Goto Y, Kurozumi A, Enokida H, Ichikawa T, Seki N (2015). Functional significance of aberrantly expressed microRNAs in prostate cancer. Int. J. Urol..

[52] Saini S (2011). Regulatory role of mir-203 in prostate cancer progression and metastasis. Clin. Cancer Res..

[53] Choi N (2015). miR-93/miR-106b/miR-375-CIC-CRABP1: a novel regulatory axis in prostate cancer progression. Oncotarget.

[54] Man YG (2011). Aberrant expression of chromogranin A, miR-146a, and miR-146b-5p in prostate structures with focally disrupted basal cell layers: an early sign of invasion and hormone-refractory cancer?. Cancer Genomics Proteomics.

[55] Zhang X, Zhang T, Yang K, Zhang M, Wang K (2016). miR-486-5p suppresses prostate cancer metastasis by targeting Snail and regulating epithelial-mesenchymal transition. Onco Targets Ther..

[56] Chen T., Guestrin C. XGBoost: A Scalable Tree Boosting System. ArXiv e-prints (2016).

[57] Wang D, Wang J, Lu M, Song F, Cui Q (2010). Inferring the human microRNA functional similarity and functional network based on microRNA-associated diseases. Bioinformatics.

[58] He T, Heidemeyer M, Ban F, Cherkasov A, Ester M (2017). SimBoost: a read-across approach for predicting drug–target binding affinities using gradient boosting machines. J. Cheminformatics.

[59] Chen T., He T. Higgs boson discovery with boosted trees. In *International Conference on High-Energy Physics and Machine Learning*, Vol. 2014, p. 69-80 (2014).

