# Supplementary material for: EGBMMDA: Extreme Gradient Boosting Machine for MiRNA-Disease Association prediction
Source: Cell Death Dis. 2018 Jan 5;9(1):3. doi: 10.1038/s41419-017-0003-x (PMC5849212; doi:10.1038/s41419-017-0003-x)
Supplement: Supplementary file 1 — Supplementary material [file 41419_2017_3_MOESM1_ESM.docx]

**EGBMMDA: Extreme Gradient Boosting Machine for MiRNA-Disease Association prediction**

Xing Chen^1,#,*^, Li Huang^2,#^, Di Xie^3^, Qi Zhao^3,4^

^1^School of Information and Control Engineering, China University of Mining and Technology, Xuzhou, 221116, China

^2^ Business Analytics Centre, National University of Singapore, 119613, Singapore

^3^School of Mathematics, Liaoning University,

Shenyang, 110036, China

^4^Research Center for Computer Simulating and Information Processing of Bio-Macromolecules of Liaoning Province, Shenyang, 110036, China

*Corresponding author

#The authors wish it to be known that, in their opinion, the first two authors should be regarded as joint First Authors.

**Email**: [xingchen@amss.ac.cn](mailto:xingchen@amss.ac.cn)

Keywords: microRNA, disease, association prediction, Extreme Gradient Boosting Machine

**Supplementary Information**

**Supplementary Table 1.** We applied EGBMMDA to prioritize all the candidate miRNA-disease pairs based on all the known miRNA-disease associations in HMDD database as training samples. This prediction result is released for further experimental validation and research.

**Supplementary Table 2**. The human miRNA-disease associations dataset used to train EGBMMDA was retrieved from the latest version of the HMDD database, covering 5430 experimentally confirmed associations between 495 miRNAs and 383 diseases.
